# Education and knowledge helps combating malaria, but not *degedege*: a cross-sectional study in Rufiji, Tanzania

**DOI:** 10.1186/1475-2875-13-200

**Published:** 2014-05-28

**Authors:** Astrid Onarheim Spjeldnæs, Andrew Y Kitua, Bjørn Blomberg

**Affiliations:** 1 National Centre for Tropical Diseases, Department of Medicine, Haukeland University Hospital, Bergen, Norway; 2 Centre for International Health, Faculty of Medicine and Dentistry, University of Bergen, Bergen, Norway; 3 National Institute for Medical Research (NIMR), Dar es Salaam, Tanzania; 4 Department of Clinical Science, University of Bergen, Bergen, Norway

**Keywords:** Malaria, Health knowledge, Attitude and practice, *degedege*, Local culture, Prevention and care, Rufiji, Tanzania, East Africa

## Abstract

**Background:**

Traditional medicine is readily available in Tanzania, and local terms like *degedege* is widely used for malaria-like illnesses, often associated with supernatural forces. Malaria prevention and intervention efforts can benefit from policy-makers’ awareness of local perceptions and beliefs in the rural areas affected by malaria. This study measured knowledge, attitudes and behaviour towards malaria and malaria-like illnesses.

**Methods:**

A cross-sectional survey was conducted in a rural area in Rufiji, Tanzania. A case report form employing a scoring system was used to capture participants’ knowledge of malaria and another for preventive actions against malaria. Logistic regression was used to assess factors associated with knowledge and preventive action.

**Results:**

Most of the participants possessed good knowledge about malaria transmission (82.1%), prevention (85.2%) and where to get treatment (96.4%). Fewer were familiar with fever (58.2%) and other common symptoms of malaria (32.7%), and even fewer actually put their knowledge into action. The action score measured the use of bed net, treatment of nets, indoor use of insecticide residual spraying (IRS), and proportion of households with tight windows, among the participants. As many as 35.7% scored zero on preventive actions, while 37.2% achieved a high action score. Education level and belonging to the age group 30 to 49 were significantly associated with higher knowledge. Education level was associated with higher score for preventive action (OR 2.3, CI 95% 1.2-1.4). Participants generally perceived *degedege*, a local name for an illness with convulsion, as different from malaria both with regards to cause and possible preventive and curative interventions.

**Conclusion:**

Respondents considered *degedege* to have supernatural causes and to need treatment by a traditional healer. This may be one reason for care-seeking shopping and care-seeking delay. Regarding *degedege* as a separate entity may explain why malaria is not perceived as a serious health problem in the area, and why little preventive actions are taken. While the elders have high status in the society, their lack of knowledge of malaria may impact the care-seeking pattern of their families.

## Background

Half of the world’s population (3.3 billion) lives in malaria-endemic areas. According to WHO, there are approximately 216 million malaria cases annually, resulting in 655 000 deaths (2010). Most of the burden and 90% of the deaths occurs in sub-Saharan Africa. Every minute a child dies because of the parasite *Plasmodium* which is transmitted to humans through bites from an infected female *Anopheles* mosquito [1]. The United Nations’ Millennium Development Goal 6C from 2000 is to "Have halted by 2015 and begin to reverse the incidence of malaria and other major diseases" [2]. Tanzania is one of the countries that has endorsed this and, with several governmental interventions, the incidence of malaria has decreased since 2000 [2]. Nevertheless, malaria remains a leading public health problem in Tanzania mainland, with an estimated 11 million annual cases and malaria contributing to 36% of all deaths among children under five years annually [3].

Increasing resistance to malaria drugs and limited access to treatment and preventive tools pose challenges to malaria control programmes. Furthermore, malaria control is dependent on consideration of sociocultural aspects and a sound understanding between the local population and policy-makers [2]. Common symptoms of malaria, such as fever, headache, muscle and joint pains, are non-specific, and are common complaints with a number of other illnesses, including both trivial and hazardous infections of viral, bacterial or parasitic aetiology. The ambiguous constellation of symptoms of malaria is unfortunate, as untreated malaria can progress quickly to a severe and fatal condition. Severe or cerebral malaria may present with additional symptoms such as anemia, respiratory distress, prostration and convulsions [1,4-6]. Many rural areas in Tanzania, including Rufiji, have explanatory models about health and illnesses and local terms that differ from the modern biomedical view held by decision-makers. The literature reveals a wide range of local terms of fever and malaria-like illnesses, and suggests that belief in witchcraft and the use of traditional medicine is very much alive in Tanzania. *Homa ya malaria* literary means ‘malaria fever’ in Kiswahili and fever is often associated with the "normal" mild malaria [7]. The term *malaria ya kawaida* is also used and means ‘common malaria’ [4,8]. Convulsions, that could indicate severe malaria, is often perceived as another disease, not related to malaria, but associated with supernatural causes, witchcraft and evil spirits [4,6-9]. These common beliefs could also have some local varieties. For instance, in a study from Tanga, in northeast coastal Tanzania, malaria was described as *one* disease with several causes, which needed different treatments in the right order. The ‘normal’ malaria should be treated with biomedical treatment, while the ‘witchcraft malaria’ required traditional medicine [6]. The population use both biomedical and traditional medicine frequently, often interchangeably, and a change in the type of treatment used is often related to change of symptoms, treatment failure or relapse [4,8,9]. While fever often is associated with malaria, convulsions tend to be associated with other ailments that have been given many names. In Tanga region the terms *uchawi*, *upepo* and *zongo* were used, but the most frequently employed terms were *degedege* and *mchango*[6] Communities near lake Victoria frequently used the terms *michango* and *nzoka*[9]. Available literature suggests that the term most widely used in Tanzania is *degedege*, which will be the focus of the current study [4,6-8]. The Tanzanian Kiswahili word *ndege* means bird, and many villagers believe that *degedege*, with convulsions reminiscent of flapping bird wings, is a carrier of a ‘bird spirit’ requiring the attention of a traditional healer [4,10-12]. Makundi and colleagues described *degedege* as a disease caused by a bird-like creatures or a moth, and other studies have associated *degedege* with supernatural forces as well as with malaria [4,10-13].

For decades, social science has highlighted the need to focus on human behavior and understanding to strengthen malaria prevention and control. While malaria programmes have improved, better utilization of findings from social research may still advance programme performance [13].

The current study measures knowledge, attitudes and behaviors towards malaria and malaria-like illnesses in order to highlight potential areas of improvements in Tanzanian malaria policy.

## Methods

### Study area and study population

The study was conducted in Rufiji, a rural district in the southern, coastal Pwani region of Tanzania. With its lowland, tropical forests, hot weather, rainy seasons and the Rufiji River’s large floodplain and delta area, Rufiji is a holo-endemic malaria area. Malaria and other febrile illnesses are among the major causes of mortality among the inhabitants. Two hospitals, five government health centers and 48 government dispensaries are distributed over an area of 14,500 square kilometers. The majority (89%) of the population lives within 5 km of one of these formal health facilities. Residents frequently buy medicines from local shops and visits traditional healers. Each village has a primary school, but 34% of the men and 66% of the women are illiterate. The main economic activity is farming of crops [14].

### Sample and study design

Rufiji has a total population of about 182,000 inhabitants and has 94 registered villages [14]. In this cross-sectional survey, 196 persons from 20 different villages were recruited by simple random sampling from August to November 2008. The randomization was performed in two stages: The names of all the villages were written on one note each, then notes with names of 20 villages were picked randomly from a hat. Thereafter, ten households (six in the last village) were selected as the researcher team walked in two different directions in each village, using the head of the village’s office (or similar center point) as a starting point. The first person that was met in the household was included in the study, provided they consented and fulfilled the inclusion criteria. If nobody was present in the household or a person was met that could not participate, the team proceeded to the next house until the required number of participants was reached. Only four persons were excluded from the study. All of them declined to participate because they thought they had to give blood, despite the team’s efforts to confirm that no blood samples were required. The respondents took part in a structured interview that intended to capture their knowledge of malaria, preventive actions, care-seeking patterns, and associations, attitudes and beliefs regarding *degedege* and various treatment options. The interviews were conducted in the participants’ own homes by three female project workers, who were native Swahili speakers with experience from similar studies.

Several studies have examined knowledge, attitude and practice (KAP) by using different scoring systems. Two new scoring systems, based on previous KAP studies [15-19] were developed and modified to fit this study.

A scoring system entitled ‘knowledge score’ was designed to capture the respondents’ knowledge of malaria. To gain full score the respondents had to: mention the most common symptom, fever (1 point); two or three other common symptoms including headache, vomiting or body pain/ache (1 point); know that malaria is transmitted by mosquitoes (1 point); know that bed nets can be used as prevention (1 point); and mention hospitals as facilities for treatment (1 point) [1]. In this study, the term hospitals referred to government and private hospitals, as well as health clinics. All together, the highest score was 5 points. High and low knowledge scores were defined as scores above and below the mean score, respectively. Data concerning *degedege* was collected by asking whether the participant was familiar with the term *degedege* and whether they could mention symptoms, causes and possible relevant preventive actions. Apart from spontaneous responses, participants were allowed to select from a list of responses compiled from other studies concerning *degedege*.

The other scoring system entitled ‘action score’ was developed to measure the preventive actions of malaria. The respondents gained full score if at least one person in the household used bed net of any kind (1 point), if the bed nets in the household were treated with insecticides within the previous six months (1 point), if the house was sprayed with insecticide residual spray (IRS) within the previous 12 months (1 point); and, if the windows were closed or covered with mosquito net (1 point). All together the highest score was 4 points. High and low scores were defined as those above and below the mean score, respectively.

### Data management and analysis

Cronbach’s Alpha test was used to assess the reliability of the two scoring systems. The resulting values, 0.70 for knowledge score and 0.74 for action score, indicated that the scoring systems had sufficient internal consistency [20]. SPSS version 18.0 and 20 were used for interpretation of the analysis. Frequencies and proportion were used for descriptive analysis, while chi-square and multivariate logistic regression were used to capture significant differences based on socio-demographic factors.

### Ethical considerations

The Regional Ethics Committee (REK) in Bergen, Norway and the National Institute of Medical Research (NIMR), Tanzania approved the study in 2008. Approval was also given from the local governmental hospital and the head of the villages of Utete, Rufiji. Only participants providing informed written consent were enrolled. All data were handled with confidentiality. Participants were free to withdraw from the study at any time.

## Results

### Sociodemographic characteristics

The participants ranged from 18 to 80 years old and 61.7% were women. More than half (65.3%) of the respondents were farmers and 79.1% lived in a house made of dung. A majority (85.2%) obtained water from a well and more than half (54.6%) were not able to pay more than 1$ for medical treatment.

### Knowledge score and action score

**Figure 1:**
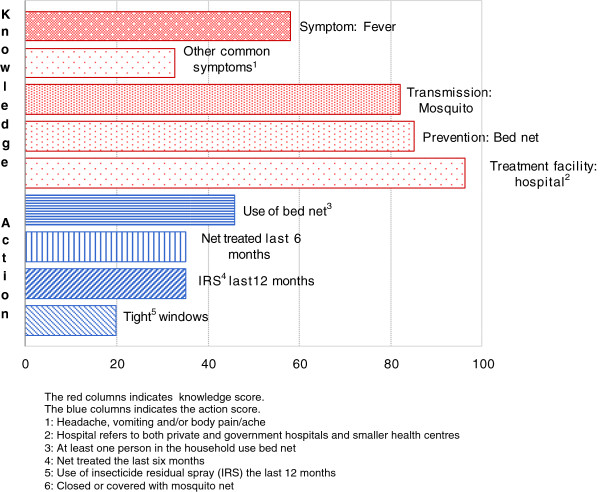
Knowledge score and action score.

The majority (82.1%) of the respondents correctly recognized mosquitoes as the agents of transmission (Figure 1), bed nets as prevention tools (85.2%) and hospitals as appropriate treatment facilities (96.4%). On the other hand, there was contradicting opinions about common malaria symptoms: slightly more than a half (58.2%) mentioned fever as a symptom of malaria, and only a third (32.7%) mentioned other common symptoms such as headache, vomiting and body pain. Altogether only 17.3% of the participants were able to achieve full score (i.e. 5 points), while 40.8% gained 4 points. The mean score was 3.55 points, corresponding to 58.2% with high knowledge score. Only 19.9% mentioned convulsions as a possible symptom.

The action score was generally lower than the knowledge score. Less than half of the participants (45.9%) said that at least one person in the household slept under a bed net (treated or untreated), slightly more than a third (35.2%) had treated their bed nets with insecticides during the previous six months, a similar proportion had used IRS during the previous year and only 19.9% had windows with mosquito-protective screens. More than a third (35.7%) achieved zero in action score, while 37.2% reached a high action score. The high score was defined by ‘above mean score’, which was 1.16. Only six persons (3.1%) among the study population achieved a full action score.

The study captured some local variations of protective methods: *fumba*, two straw ‘blankets’ wrapped around the body, was mentioned as protection against malaria among 9.7% of the study population. *Mwarobaini*, a tree believed to treat 40 diseases is a well-known herbal medicinal plant in many areas in Tanzania, including Rufiji, and 12.2% of the participants mentioned this as a treatment for malaria [21].

By logistic regressions (Table 1), belonging to the age group 30 to 49 and having primary education were significantly associated with a high knowledge score (odds ratio (OR) 3.1, 95% confidence interval (CI) 1.3-7.5, and OR 2.9, 95% CI 1.3-6.3, respectively). Furthermore, primary and middle/higher education was associated with high action score (OR: 7.1, 95% CI 2.4-20.9 and OR 27.4, 95% CI 5.6-135.5, respectively). Only a few of the participants had middle/higher education and this was not significantly associated with higher knowledge or action score. Those aged 50–80 years had a significantly lower action score than the younger inhabitants in the area, aged 18–29.

**Table 1:** Knowledge score and Action score depending on background factors, univariate and logistic regression

**Background factors**	**High score**		**Univariate analysis**		**Multiple response/Logistic regression**	
	**% (n)**		**OR (95% C.I.)**		**OR (95% C.I.)**	
	**Knowledge score**	**Action score**	**Knowledge score**	**Action score**	**Knowledge score**	**Action score**
**Sex**						
Male (75)	56.0 (42)	32.0 (24)				
Female (121)	59.5 (72)	40.5 (49)	1.2 (0.7 – 2.1)	1.5 (0.8 – 2.7)	1.7 (0.8 – 3.4)	1.7 (0.8 – 3.8)
**Age**						
18 – 29 (58)	56.9 (33)	44.8 (26)				
30 – 49 (93)	68.8 (64)	41.9 (39)	1.7 (0.9 – 3.3)	0.9 (0.5 – 1.7)	3.1 (1.3 – 7.5)^a^	1.1 (0.5 - 2.8)
50 – 80 (45)	37.8 (17)	17.8 (8)	0.5 (0.2 – 1.0)	0.3 (0.1 – 0.7)^b^	1.2 (0.4 – 3.8)	0.5 (0.1 – 1.6)
**Education**						
None (47)	34.0 (16)	10.6 (5)				
Primary school (130)	65.4 (85)	43.1 (56)	3.4 (1.7 – 6.9)^c^	6.5 (2.4 – 17.5)^c^	2.9 (1.3 – 6.3)^b^	7.1(2.4 – 20.9)^c^
Middle/high (18)	66.7 (12)	66.7 (12)	3.9 (1.2 – 12.3)^a^	16.8 (4.4 – 64.8)^c^	3.0 (0.8 – 11.4)	27.4 (5.6 – 135.5)^c^
**Marital status**						
Ever married (156)	55.8 (87)	36.5 (57)				
Not married (40)	67.5 (27)	40.0 (16)	1.7 (0.8 – 3.4)	1.2 (0.8 – 2.4)	1.8 (0.7 – 4.9)	0.5 (0.2 – 1.2)
**Occupation**						
Farmer (128)	56.2 (72)	32.8 (42)				
Housewives (15)	60.0 (9)	53.3 (8)	1.2 (0.4 – 3.5)	2.3 (0.8 – 6.9)	1.1 (0.3 – 3.9)	2.5 (0.7 – 9.0)
Selfemployed (16)	62.5 (10)	56.2 (9)	1.3 (0.4 – 3.8)	2.6 (0.9 – 7.6)	1.1 (0.3 – 3.6)	2.8 (0.8 – 9.8)
Other* (31)	62.2 (23)	37.8 (14)	1.3 (0.6 – 2.7)	1.3 (0.6 – 2.7)	1.1 (0.5 – 2.8)	0.8 (0.3 – 2.1)
**In household**^**#**^						
No CU5** (75)	54.7 (41)	33.3 (25)				
CU5** (121)	60.3 (73)	39.7 (48)	1.3 (0.7 – 2.3)	1.3 (0.7 – 2.4)	0.8 (0.4 – 1.7)	0.9 (0.4 – 2.0)
No pregnant women (129)	54.3 (70)	38.0 (49)				
Pregnant women (67)	65.7 (44)	35.8 (24)	1.6 (0.9 – 3.0)	0.9 (0.5 – 1.7)	1.8 (0.9 – 3.7)	0.7 (0.3 – 1.4)
Malaria not experienced^##^ (125)	53.6 (67)	33.6 (42)				
Malaria experienced^##^ (71)	66.2 (47)	43.7 (31)	1.7 (0.9 – 3.1)	1.5 (0.8 – 2.8)	1.9 (1.0 – 3.8)	1.3 (0.6 – 2.7)
**Total**	58.2 (114)	37.2 (73)				

^a^p.value < 0.05 ^b^p.value < 0.01 ^c^p.value < 0.001.

*No CU5* means that there were no children under five years in that particular household.

*No pregnant women* means that there was no pregnant women in that particular household.

*i.e.: nurse, traditional healer, driver, artist, student.

**Children under five years.

^#^Refers to at least one person in the household within each group.

^##^Experienced the last six months.

Overall, a high knowledge score was significantly associated with a high action score (OR 2.3, 95% CI 1.2-4.1). There was no association between high knowledge score and having someone in the household using bed nets (OR: 1.3, 95% CI 0.71-2.22). There was also no association between high knowledge score and the use of IRS (OR 1.5, 95% CI 0.68-3.47). However, high knowledge score was statistically associated with having bed nets treated with insecticide recently (OR 2.9, 95% CI 1.5-5.5) and having mosquito-protective screens on the windows (OR 2.5, 95% CI 1.1-5.4).

### Familiarity with *degedege*

A majority (69.9%) of the respondents were familiar with the term *degedege*. Convulsion was the most (54.6%) frequently mentioned symptom, followed by neck stiffness (18.4%) and tiredness/weakness (9.7%). A small percentage mentioned fever and changes in the eyes (both 7.7%). More than half could not mention any cause of *degedege* or how to protect themselves from this (55.1 and 56.1%, respectively). Nevertheless mosquitoes (12.2%), spirits (11.2%) and high fever (9.7%) were mentioned as probable causes, while medicines (without specifications) (12.8%), bed net (11.7%) and vaccination (8.2%) were mentioned as possible preventions.

### Use of bed nets

**Figure 2:**
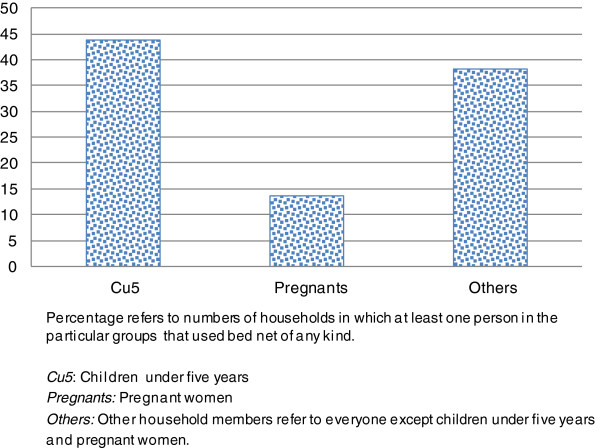
Use of bed net in household among children under five years, pregnant women and others.

A majority (73.0%) of the respondents possessed at least one bed net of any kind in their household, but only 62.9% of these were in use. As shown in Figure 2, less than half (43.8%) of children under five years only 13.6% of pregnant women and 38.3% of other groups in the households, slept under a bed net. Further assessment revealed that neither pregnant nor others in these particular households used bed nets.

Knowing the transmission of malaria or having experienced malaria recently did not increase the use of bed nets in the household (OR 0.66, 95% CI 0.32-1.38 and OR 1.62, 95% CI 0.90-2.91, respectively).

### Care-seeking patterns

A majority of the households (80.6%) had experienced that someone in the household had been seeking some kind of care the last six months. Among these, almost a half (46.8%) decided to seek care at the hospital only. Approximately an equal proportion (46.2%) opted for multiple care seeking, including a combination of hospitals, traditional healers and/or treatment at home. Only a few (7.0%) used exclusively a treatment option outside a formal hospital.

**Figure 3:**
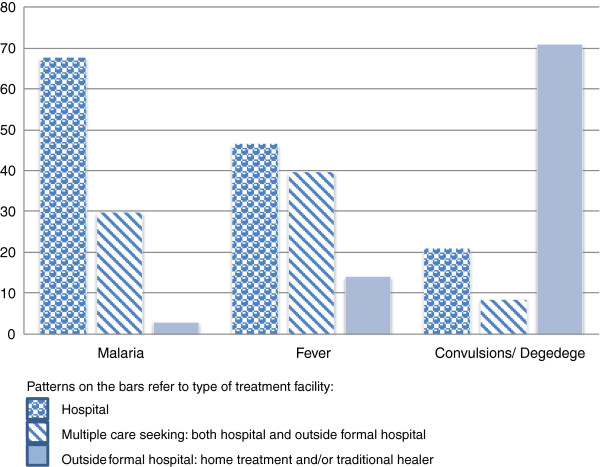
Care-seeking depending on perceived illness or disease.

Among all respondents, 36.2% had experienced malaria, while 51.5% had experienced fever and 12.2% reported having experienced convulsions or what they perceived as *degedege* in their household within the last six months.Figure 3 brings up disparities concerning care-seeking patterns for different conditions. Hospitals appeared to be a common choice when suffering from malaria (67.6%), but at the same time almost a third (29.6%) chose to seek care multiple places for the same disease. When the participants suffered from fever, 46.5% preferred to seek care at the hospital, while 39.6% selected several options. Few of those suffering from malaria (2.8%) or fever (13.9%) decided to use exclusively treatment outside a formal hospital.

Suffering from convulsions or *degedege* seemed to initiate different actions, as a majority (70.8%) sought help outside the formal health service sector, and only 20.8% decided to attend a hospital. A few respondents (8.3%) opted to go for multiple treatment options when suffering from convulsions or *degedege*.

### Opinions regarding treatment options

In general, those who had received some kind of care were very satisfied with how they were treated, regardless of whether they had been at a hospital, a traditional healer or had used home treatment (92.4, 89.5 and 79.4%, respectively). In addition, the majority (93.4%) reported that they had great confidence in hospital-based treatment. While almost a third (31.6%) had great trust in traditional healers, a fifth (20.4%) did not and almost a half (46.9%) of the respondents did not have any clear opinion on this. Almost a third (29.5%) trusted home treatment. The majority (90.8%) claimed that they would follow the advice given at the hospital, while almost a quarter (24.0%) would follow advice from a traditional healer.

## Discussion

This study assessed knowledge and preventive actions against malaria, local understanding of *degedege*, care-seeking patterns and opinions regarding various treatment options in order to highlight potential areas for improvements to malaria control programmes. The study identified a gap between the study population’s knowledge about malaria and the actual preventive and care-seeking actions taken in response to perceived illness, as well as a divergence between opinions and actual behaviour regarding various treatment options.

### Gap between knowledge and action

The findings indicate that most respondents possess adequate knowledge about malaria prevention and treatment. However, the study identified a major challenge as certain symptoms, such as convulsions, which may signify severe malaria or other severe infections, did not seem to prompt the study population to seek help from the public health system. Rather, respondents seem to feel that symptoms such as convulsions are associated with *degedege* and need the attention of traditional healers. One of the major obstacles in combatting malaria is that malaria cannot be diagnosed based on symptoms alone, even by experienced clinicians and much less by the general population. In the study area there is still an obvious need to promote knowledge to promptly seek care at a medical facility for malaria screening and possible treatment when people have symptoms that could represent malaria, such as fever, headache, and febrile convulsions, which may be associated with severe malaria.

The association between education and knowledge is consistent with results from a study from Guatemala [17] where literates had significant higher scores than illiterates, while other studies from sub-Saharan Africa have shown conflicting results [16]. Illiteracy excludes access to written information, whereas education may help the understanding of abstract relationships and explanations for diseases and may improve ability to evaluate information and explanations.

The age group 30–49 had the highest knowledge scores. A reason for this could be that they have more life experience than the youngest age group, while, but also have been exposed more to up-to-date health information than the older generation. Many in the 30–49 years group would have experienced the introduction of bed nets at discounted prices for pregnant women through the Tanzanian National Voucher Scheme, *Hati Punguzo*[22,23]. Starting in 2004 it was expanded in order to include infants in 2006. The campaign covered 75% of Pwani Region, and Rufiji was one of the districts pointed out to receive free bed nets to children under five years of age through a child health campaign in 2005 [24]. Even though findings showed that few pregnant women and less than half of children under five years used bed nets in the study population, this campaign could have influenced the knowledge among the age group 30–49 in a positive direction.

The age-group 50–80 years, which obtained low knowledge and action scores, belongs to a generation that has not benefitted from free bed net campaigns and other public health campaigns. The older generation, remembering a history with failing control programmes may, understandably, have lower trust in the public health system’s advice and may thus rely more strongly on traditional health care than modern biomedicine [25].

Contrary to the study from Guatemala [17], where knowledge was increased among those with personal experience with malaria, the current study found no difference in knowledge score, action score and the use of bed nets among those who had experienced malaria recently and those who had not. A reason could be that those who had experienced malaria were not familiar with malaria transmission and therefore did not know how they were infected or how to protect themselves. A possible reason for this could be that the information at the treatment facility was inadequate or not compelling enough. Furthermore, they may have suffered from mild malaria attacks with few complications, leading to the impression that it is better to suffer occasionally from malaria than to sleep constantly in the uncomfortable heat underneath a bed net.

Overall, findings showed a gap between knowledge and preventive actions among the respondents. Many inhabitants in Rufiji have sufficient knowledge to avoid malaria and access treatment in the appropriate facilities, but nevertheless opt not to use this knowledge. An explanation for this mismatch might be that information on the consequences of malaria may not have had much impact on the population in rural Rufiji. The same tendency was seen in Swaziland [16], where most of the respondents knew how to protect themselves, but chose not to. A reasonable explanation could be lack of preventive materials, but knowing that only 62.9% of all bed nets among the respondents were in use this can only partially explain the lack of preventive actions. According to Pulford *et al.*[26], reasons for not using bed nets are concerns regarding discomfort, technical issues, perceived mosquito density and availability. Discomfort due to high ambient temperature, is likely to be a reason that applies to inhabitants of the warm climate in Rufiji, especially among pregnant women who barely used bed nets. This can have serious consequences in areas such as Rufiji, which are holo-endemic for malaria [14]. Conversely, less use of bed nets during the hottest season may have less impact in some areas since the density of mosquitoes tends to lower. Technical issues such as attaching bed nets in houses made of dung are also a possible explanation why not all bed nets are in use.

Misconceptions about malaria may be an important reason for a gap between knowledge and action, as previously suggested by Maslove *et al*. [27]. Findings showed that a majority of the study respondents was familiar with symptoms of *degedege*, where convulsions were mentioned most frequently. Convulsions could be a symptom of several diseases including various forms of epilepsy, and the differential diagnoses for fever-associated convulsions include meningitis, encephalitis and other infections including severe malaria, as well as simple febrile seizures secondary to any infection that produces fever in a child. It is therefore not possible to conclude that *degedege* is synonymous with malaria, but rather a local name for several conditions, where convulsions appear as the most prominent symptom. Few respondents mentioned fever and mosquitoes in the context of *degedege*, and also few mentioned convulsions in association with malaria, even though it is a frequent symptom of severe malaria/cerebral malaria in children [1,28]. These findings indicate that malaria is perceived as a mild disease, whilst *degedege*, probably encompassing both severe malaria and a number of other conditions, is perceived as a serious threat.

The relationship between malaria and the concept of *degedege* has been highlighted in several studies [10,29], sometimes described as two separate illnesses, while some studies found that convulsions, the hallmark of *degedege*, were recognized as the symptom separating severe malaria from mild malaria. In Rufiji, a region where people suffer from poverty, lack of food and clean water and high burden of HIV, tuberculosis, birth complications, childhood diarrhoea etc., malaria is only one of many challenges that must be combated daily.

### Care-seeking patterns based on perceived illness/disease

The study findings suggest that multiple care seeking is common among the study population. An important finding is that care-seeking patterns change towards the traditional health sector when respondents suffer from convulsions or from what they perceived as *degedege*. This is comparable to other studies where care-seeking was modified when symptoms changed [29,30]. Some studies found that *degedege* was perceived to be associated with supernatural forces requiring a traditional healer, while others found that *degedege* needs timely attention at modern treatment facilities, indicating that traditional treatment is not an important factor for care-seeking delay any more [10,29,31]. These contradicting findings from neighbouring areas of Rufiji indicate that there may be several local differences in behaviour and beliefs within districts. This suggests that local differences must be considered to combat malaria.

Caretakers of children, generally mothers, are not necessarily the decision-makers in the household. The typical Tanzanian rural community is patriarchal with the decision-maker of the household often being an elderly man [29,32]. Many health interventions focus on educating the mothers, but even when a mother has sound knowledge about malaria she does not necessarily have the funds and means to take the final decision about when and where to access treatment [33]. Many women must wait for the endorsement of the decision-maker [30,34], which could cause delays in Rufiji, where many men work in the field away from home [24]. The older generation in this study scored lower on knowledge about malaria, and they may therefore recommend treatments that are not consistent with the modern biomedical view. Studies have also shown that women have experienced low sympathy from health workers when showing up late for treatment, leading to even more disempowerment and discouragement [32]. Such attitude towards young women could contribute to delaying care-seeking. The social gap between the local rural population and the expert decision-makers may be large in many countries in sub-Saharan Africa, where only a fraction of the population has access to higher education [32].

### Opinions towards various treatment options

Most of the respondents were very satisfied with the treatment regardless of which facility they had visited. A great trust in the hospital as a treatment facility may reflect that the questions were asked by representatives from a biomedical research community, but still a third admitted that they have great trust in traditional healers. The study may be biased by respondents’ unwillingness to disclose their actual opinion while facing obvious representatives of the public health system. Even fewer would follow advice from a traditional healer or advice given in relation to home treatment. In other studies [29] people were unwilling to talk about traditional medicine in fear of negative reactions from health workers.

Although the respondents expressed more confidence in hospitals, they also frequently visited traditional healers. Living within a distance of 5 km from a hospital, as is the case for the majority in Rufiji [14], does not imply that the hospital is readily accessible. Even short distances on poor roads or without any means of transportation is challenging for a sick person, especially during the rainy seasons. Traditional healers are usually geographically nearer to the patients and more accessible at any time, night and day [29].

As observed by Makundi *et al*. [4], many healers are respected, not only because of their knowledge and age, but also because of their kindness and way of communicating with the local population. Even though it was evident that most of the study population understood hospitals may be the best treatment facilities for malaria, visiting the traditional healer appears to be important in order to express respect and to obtain comprehensible information using more familiar explanatory models.

The study has a number of limitations. The sample size was limited in number and geographical location. The cross-sectional design is limited to describing associations between variables at one time, while a longitudinal design would have allowed for better follow-up the participants over time. The study carried a risk of misinterpretations of the language, but care was taken to use a research team fluent in Kiswahili, the major Tanzanian language. Categorization of the participants’ responses was necessary for data management issues and for statistical testing, but may also have concealed subtle aspects of the respondents’ answers.

## Conclusions

The study population in Rufiji is not passive receivers of health care, but actively seek care among available providers. Many factors appear influential on the final decision of preventive actions and treatment choice, including local beliefs, sociocultural traditions and accessibility. This study underlines the continued need to take into account the local social and contextual factors in which the intervention programmes are being implemented.

This study identified that elders hold the lowest knowledge about malaria among the study population, and could be an important barrier for both prevention and care-seeking behaviour, because of the respect the population devote to elders. The elders may keep alive old traditions and beliefs that could be contradictory to modern biomedical advices. Involving the elders of Rufiji in decision-making in new health programmes may be valuable, not only to increase their knowledge, but also provide an opportunity for them to share their knowledge in a useful way. Empowerment of the local population, especially the older generation, could improve cooperation and facilitate behaviour change. Behaviour change obtained on the local population’s own terms may be more sustainable. Focusing on educating caretakers when they are disempowered and not in the position to use their knowledge may not help prevent malaria, unless the decision-makers and the elderly men are also included in the educating programme.

In Rufiji, many inhabitants are illiterate. While education is the long-term solution, visual communication, such as lectures, drama, film, and photos, has proved to be helpful elsewhere in Tanzania [35,36].

## Competing interests

The authors declare that they have no competing interests.

## Authors’ contributions

AOS conceived and designed the study, did the fieldwork and data management, and led the analysis and interpretation of the data and writing of this paper. AYK and BB contributed to the study design, the analysis and writing of the paper. All authors read and approved the final manuscript.
